# Effects of Cognitive Reserve in Alzheimer’s Disease and Cognitively Unimpaired Individuals

**DOI:** 10.3389/fnagi.2021.784054

**Published:** 2022-02-07

**Authors:** Dong Hyuk Lee, Sang Won Seo, Jee Hoon Roh, Minyoung Oh, Jungsu S. Oh, Seung Jun Oh, Jae Seung Kim, Yong Jeong

**Affiliations:** ^1^Graduate School of Medical Science and Engineering, Korea Advanced Institute of Science and Technology, Daejeon, South Korea; ^2^College of Korean Medicine, Sangji University, Wonju, South Korea; ^3^Research Institute of Korean Medicine, Sangji University, Wonju, South Korea; ^4^Department of Neurology, Samsung Medical Center, Sungkyunkwan University School of Medicine, Seoul, South Korea; ^5^Department of Physiology, Korea University College of Medicine, Seoul, South Korea; ^6^Neuroscience Research Institute, Korea University College of Medicine, Seoul, South Korea; ^7^Department of Nuclear Medicine, Asan Medical Center, University of Ulsan College of Medicine, Seoul, South Korea; ^8^Department of Bio and Brain Engineering, Korea Advanced Institute of Science and Technology, Daejeon, South Korea; ^9^KI for Health Science and Technology, Korea Advanced Institute of Science and Technology, Daejeon, South Korea

**Keywords:** cognitive reserve, Alzheimer’s disease, AD spectrum, cognitive aging, multimodal neuroimaging

## Abstract

The concept of cognitive reserve (CR) has been proposed as a protective factor that modifies the effect of brain pathology on cognitive performance. It has been characterized through CR proxies; however, they have intrinsic limitations. In this study, we utilized two different datasets containing tau, amyloid PET, and T1 magnetic resonance imaging. First, 91 Alzheimer’s disease (AD) continuum subjects were included from Alzheimer’s Disease Neuroimaging Initiative 3. CR was conceptualized as the residual between actual cognition and estimated cognition based on amyloid, tau, and neurodegeneration. The proposed marker was tested by the correlation with CR proxy and modulation of brain pathology effects on cognitive function. Second, longitudinal data of baseline 53 AD spectrum and 34 cognitively unimpaired (CU) participants in the MEMORI dataset were analyzed. CR marker was evaluated for the association with disease conversion rate and clinical progression. Applying our multimodal CR model, this study demonstrates the differential effect of CR on clinical progression according to the disease status and the modulating effect on the relationship between brain pathology and cognition. The proposed marker was associated with years of education and modulated the effect of pathological burden on cognitive performance in the AD spectrum. Longitudinally, higher CR marker was associated with lower disease conversion rate among prodromal AD and CU individuals. Higher CR marker was related to exacerbated cognitive decline in the AD spectrum; however, it was associated with a mitigated decline in CU individuals. These results provide evidence that CR may affect the clinical progression differentially depending on the disease status.

## Introduction

The neuropathological hallmarks of Alzheimer’s disease (AD) are intracellular neurofibrillary tangles of hyper-phosphorylated tau protein and extracellular depositions of β-amyloid as the main component of senile plaque (Serrano-Pozo et al., 2011). The National Institute on Aging and Alzheimer’s Association (NIA-AA) recently announced a new research framework for the biological definition of AD, which focused on the diagnosis of AD with three biomarkers (Jack et al., 2018). The biomarkers were grouped into β-amyloid, tau protein, and neurodegeneration (A/T/N), capturing the overall neuropathology of AD.

Cognitive reserve (CR) stems from the discrepancy between the degree of brain pathology and its clinical manifestations (Katzman et al., 1988). The reserve concept accounts for individual susceptibility to age-related brain changes or AD-related brain neuropathology (Stern, 2012). CR is measured using surrogate markers of lifestyle, although they have several innate shortcomings (Zahodne et al., 2013). We previously demonstrated that the model reflecting overall AD neuropathology (A-T-N) could capture the properties of CR in a cross-sectional study (Lee et al., 2019).

The association between CR and clinical progression remains controversial. Several studies have reported that CR is associated with clinical progression (Bracco et al., 2007; Reed et al., 2010; Myung et al., 2017; Robitaille et al., 2018), but others did not detect an association (Singh-Manoux et al., 2011; Cadar et al., 2015; Reijs et al., 2017). These conflicting results may be due to the usage of erratic CR proxies, misdiagnosis of pure AD without AD biomarkers, and mixture of disease and unimpaired groups in the analysis.

Thus, in this study, we examined the effect of CR on the relationship between brain pathology and cognitive function and on the clinical progression over time by (1) applying a CR model capturing overall AD neuropathology, (2) selecting amyloid-positive subjects, and (3) subdividing subjects based on the disease status. We hypothesized that CR would modulate the association between brain pathology and cognition and differentially influence the clinical progression according to the disease status.

## Materials and Methods

### Participants

First, participants were recruited within the Alzheimer’s Disease Neuroimaging Initiative (ADNI 3, ClinicalTrials.gov ID: NCT02854033). Inclusion criteria for AD, amnestic mild cognitive impairment (aMCI), and cognitively unimpaired subjects (CU) followed the protocol of ADNI 3 (Number: ATRI-001). We only included amyloid-positive subjects who had all three imaging modalities [tau (^18^F-AV-1451 PET), amyloid PET (^18^F-AV-45 or ^18^F-florbetaben PET), and T1-weighted magnetic resonance imaging (MRI)]. Amyloid (Aβ) status was determined by the global standard uptake value ratio (SUVR) > 1.11 (^18^F-AV-45) or > 1.08 (^18^F-florbetaben). At baseline, the final participants comprised 25 AD, 42 prodromal AD [Aβ (+) aMCI], and 24 preclinical AD [Aβ (+) CU]. Subsequently, 77 subjects (19 AD, 36 MCI, and 22 CU) were available in the longitudinal analysis.

Second, subjects were recruited at the memory disorder clinic in the Department of Neurology at the Asan Medical Center (AMC) and the Samsung Medical Center (SMC) in Seoul, South Korea. We obtained all three modalities for each subject at baseline (tau PET [^18^THK-5351], Aβ PET [^18^F-florbetaben], and T1 MRI). All AD subjects fulfilled the clinical diagnostic criteria for AD according to the NINCDS/ADRDA, and those with aMCI met the Petersen’s criteria. Subjects with AD and aMCI were Aβ-positive as determined by brain amyloid plaque load (BAPL score) ≥ 2. CU was defined as being elderly and free of neurological disease, Clinical Dementia Rating (CDR) 0 and Mini-Mental State Examination (MMSE) > 27. At baseline, 87 participants [21 AD, 32 prodromal AD, and 34 Aβ (-) CU] were included. At 4-year follow-up, 61 and 43 subjects had available longitudinal data for the MMSE and other cognitive scores, respectively.

For dataset 1, ethics approval was obtained by the ADNI investigators. All study participants provided written informed consent. ADNI 3 is listed in the ClinicalTrials.gov registry (identifier: NCT02854033). For dataset 2, the Institutional Review Board of both hospitals approved the study, and all subjects provided written informed consent.

### Neuropsychological Assessment and Cognitive Reserve Surrogate Marker

In dataset 1, we utilized composite scores of memory (ADNI-MEM), executive function (ADNI-EF), language function (ADNI-LAN), and visuospatial function (ADNI-VIS) in ADNI 3 to construct CR marker. These were composite *z*-scores from the ADNI neuropsychological battery of test. We applied the total summation of four domain scores as a global cognitive composite score. Other cognitive measures, including the Alzheimer’s Disease Assessment Scale-Cognitive Subscale (ADAS-cog) 11 and MMSE, were used for further validation. In dataset 2, we measured the global composite score for the CR marker based on previous studies (Jahng et al., 2015; Lee et al., 2019). The global composite score was obtained from the average of five domains containing 14 neuropsychological tests (e.g., attention, visuospatial, language, executive, and memory function). The tests were composed of Seoul Verbal Learning Test (SVLT-E) and Rey Complex Figure Test (RCFT) in memory domain contrasting program, Go-No-Go test and Controlled Oral Ward Association Test (COWAT) in executive function, forward and backward Digit Span Test (DST) in Attention, RCFT copying task in Visuospatial function, and Korean-Boston Naming Test (K-BNT) in language function.

We used years of education as a CR proxy. In dataset 1, we correlated the CR marker with years of education. To further validate our CR marker, education was applied to identify CR-related regions in Aβ pathology and neurodegeneration.

### Image Acquisition

In ADNI dataset, MRI scans were acquired on scanners from different manufacturers (Philips, Best, Netherlands; GE, Cleveland, OH; and Siemens, Malvern, PA, United States) using harmonized protocols. We obtained 3D T1-weighted magnetization-prepared rapid gradient echo (MPRAGE) sequence, with 1 mm isotropic voxel resolution and repetition time (TR) = 2,300 ms. Aβ PET scans were recorded for 20 min at 50 min after 370 MBq ± 10% injection (^18^F-AV-45) or 90 min after 300 MBq ± 10% injection (^18^F-florbetaben) of the tracer. Tau PET scans were acquired for 30 min at 75–105 min after 370 MBq ± 10% tracer injection. In dataset 2, we obtained 3D T1-weighted MPRAGE with voxel size (AMC: 1.11 × 1.11 × 1.2 mm^3^, SMC: 1.0 × 1.0 × 0.5 mm^3^) and a TR (AMC: 6.8 ms, SMC: 9.9 ms) from the manufacturer Philips (Eindhoven, Netherlands). Tau PET scans were acquired for 20 min, commencing 50 min after the injection of 185 MBq ± 10% of ^18^F-THK5351. Aβ PET scans were acquired for 20 min, commencing 90 min after the injection of 300 MBq ± 10% of the tracer. Hoffman phantom-based PET harmonization was applied in datasets 1 and 2.

### Data Preprocessing

In both datasets, individual tau and Aβ PET scans were co-registered onto the individual T1 image and normalized into Montreal Neurological Institute (MNI) standard space. Preprocessed images were smoothed (6 mm). SUVR images were calculated for all individuals using the cerebellar gray matter as a reference. All preprocessing was conducted using SPM12 (Wellcome Trust Centre for Neuroimaging, University College London) and MATLAB R2014b (The Mathworks, Natick, MA, United States). For cortical thickness, T1-weighted images underwent preprocessing steps with Freesurfer 6.0^1^. The total intracranial volume (TIV) was obtained by summing the volumes of gray matter, white matter, and CSF.

### Calculation of the Cognitive Reserve Marker

We conceptualized CR as the residual of actual cognitive performance and estimated performance; the latter was estimated from AD neuropathology, demographics, a genetic factor (ApoE ε4), and TIV. The “residual” concept for quantifying CR has been applied previously (Reed et al., 2010; Habeck et al., 2017; van Loenhoud et al., 2017). While these studies captured only the structural aspects, our method had an advantage in that the overall AD neuropathology was reflected in the model as follows (A-T-N: primary components of AD biomarkers):

Cognitive function_estimated_ = β_0_ + β_1_ × *X*_Tau_ + β_2_ × *X*_A__β_ + β_3_ × *X*_Thickness_ + β_4_ × *X*_*Age*_ + β_5_ × *X*_Sex_ + β_6_ × *X*_ApoEε__4_ + β_7_ × *X*_TIV_

(+ β_8_ × *X*_Aβ_ _tracer type_: In dataset 1, Aβ tracer type was included; *X*_Tau_, *X*_Aβ_, and *X*_Thickness_: Global value obtained from each imaging modality)

Cognitive function: Cognitive composite score using multiple domains

Cognitive reserve (CR) = Cognitive function_observed_ – Cognitive function_estimated_

As a global value for each AD pathology, we extracted the global extent of tau, Aβ, and cortical thickness per subject using multimodal imaging. In tau PET, we obtained images with GM probability > 0.5 and calculated the average tau SUVR value per subject in Braak regions of interest (ROIs) (Hoenig et al., 2017). To avoid off-target effects of the tracer, we excluded ROIs in the basal ganglia and thalamus (Harada et al., 2016). In Aβ PET, we obtained images with GM probability > 0.5 and extracted a global Aβ SUVR value in combined ROIs (Jack et al., 2010). Similarly, we measured the average thickness value among 68 Desikan ROIs. Age (Guerreiro and Bras, 2015) and sex (Mielke, 2018) are demographic risk factors for AD, and ApoE allele 4 (Liu et al., 2013; Safieh et al., 2019) is considered a representative genetic factor of AD. TIV was added as a covariate for thickness and an estimate of brain reserve (Tate et al., 2011). We subsequently performed multiple linear regression with the dependent variable of cognitive function; predictors of global tau, Aβ deposition, and thickness (AD neuropathology); and covariates of age, sex, ApoE ε4 status, and TIV. The beta coefficients of these variables were applied to estimate the cognitive function. Finally, we calculated the CR marker as the residual between the actual and estimated score. According to the equation, higher CR marker value denoted greater CR as it indicated that relatively high cognitive function was maintained at a given level of AD neuropathology in the population.

### Effect of the Cognitive Reserve Marker on the Relationship Between Pathological Burden and Cognition

For dataset 1, we calculated Pearson’s correlation between the CR marker and a conventional CR proxy, years of education. We also performed multiple linear regression using education as an outcome; CR marker as a predictor; and age, sex, and TIV as covariates.

Fundamentally, we investigated whether the CR marker could modulate the effect of brain pathology on cognitive function across the AD spectrum. To identify regions for which greater education enabled subjects to tolerate greater Aβ burden (CR-related regions), we first performed voxel-wise multiple linear regression of Aβ SUVR with education as the predictor, adjusting for age, sex, Aβ scanner type, and MMSE. The resulting *t* map was thresholded at the voxel level at α = 0.01 and corrected at the cluster level at FWE α < 0.05. We then extracted the average Aβ SUVR within the CR-related regions for each subject. Finally, we tested the interaction of the CR marker × Aβ SUVR on cognition, controlling for age and sex across the AD spectrum. We conducted similar procedures with cortical thickness in ROI-wise, adjusting for age, sex, MMSE, and TIV among the AD spectrum (FDR *p* < 0.05). We measured the atrophy value as the reciprocal of thickness value within significant regions. We tested the interaction of the CR marker × atrophy value on cognitive function, controlling for age, sex, and TIV across the AD spectrum.

### Effect of the Cognitive Reserve Marker on Clinical Progression

For dataset 2, we performed longitudinal analysis to verify the effect of the CR marker on clinical progression. For a 4-year period, we examined the conversion rate and change in cognitive scores and disease severity across the entire group. We performed Cox-proportional hazard regression among prodromal AD (*n* = 32) and CU (*n* = 34) to investigate the relationship between the CR marker and disease conversion. In this case, we calculated the CR marker without AD subjects at baseline. Diagnostic changes to more severe stages were only considered as a disease conversion. The model contained conversion with time as an outcome measure; CR marker (continuous or binary) as a predictor; and age, sex, ApoE, and TIV as covariates. We validated the effect of the CR marker by confirming hazard ratio (HR), *p*-value, and 95% confidence intervals (CIs) of HR in the model. Finally, we conducted a likelihood ratio test to evaluate the goodness of model fit between two competing models with and without the CR marker, respectively. We also assessed the Akaike Information Criterion (AIC) to estimate the relative quality of statistical models for a given data. AIC deals with the tradeoff between the goodness of model fit and complexity of the data; smaller AIC indicates higher quality (Vrieze, 2012).

We conducted linear mixed models with cognitive composite score, memory score, and MMSE as outcomes; the CR marker at baseline, time, and CR marker × time as predictors; and adjusted for age, sex, ApoE, and TIV. We repeated a linear mixed model with CDR-SB as an outcome with the same predictors and covariates. Our interest was the interaction of the CR marker and time, as this referred to the effect of the CR marker on clinical progression.

These analyses were divided into AD spectrum and CU groups, as CR behavioral pattern would exert differently according to the disease status. The 95% CIs of beta coefficient of the CR marker, time, and their interaction were estimated. Finally, we performed a likelihood ratio test and AIC to estimate the additional value of the model with the interaction.

In order to estimate more reliable CIs for beta values of the interaction in clinical progression, semi-parametric bootstrapping of the linear mixed model was performed. For this process, we made 1,000 bootstrap samples by resampling the original data with replacement and calculated 95% CIs of the bootstrapped coefficients.

For dataset 1, we repeated linear mixed models with ADAS-cog 11 as outcomes; the CR marker at baseline, time, and CR marker × time as predictors; and adjusted for age, sex, ApoE, and TIV. The analyses were also divided into AD spectrum and CU groups. We conducted a likelihood ratio test and AIC to estimate the additional value of the model with the interaction.

### Sensitivity Analysis

In a longitudinal analysis of dataset 2, to figure out the difference between using continuous Aβ SUVR value and using visual diagnosis of Aβ, first we applied binary value (positivity, negativity) instead of continuous value in the calculation of the CR marker. Then, we repeated linear mixed model analysis and compared the results with the model constructed from continuous Aβ SUVR.

Second, we investigated the effect of education on clinical progression in dataset 2. Therefore, we conducted linear mixed model analysis by replacing the CR marker with education into the model.

Finally, we added the global pathological burden (global tau, Aβ, and thickness value) in the linear mixed model as covariates to partly account for the explanation that the CR marker is only a derivative of pathological measurement in relation to cognitive function.

### Statistical Analysis

Statistical analyses were conducted in SPSS 18 (Chicago, IL, United States). To compare participant characteristics, chi-square tests and ANOVA tests were performed. In the longitudinal analysis, linear mixed model analysis and semi-parametric bootstrapping were conducted using R^2^.

## Results

### Participant Characteristics

As shown in Table 1, we observed significant differences among the three groups in global tau, Aβ deposition, cortical thickness, and cognitive scores. In dataset 1, we investigated the A/T/N classification status using CSF information. Among the 91 subjects, 62 subjects (16 AD, 26 MCI, 20 CU) have baseline CSF information of p-tau181 and t-tau. Unlike the pathological burden obtained from imaging modalities, CSF levels of p-tau and t-tau did not differ significantly among the three groups (Table 1). Abnormal levels of CSF biomarkers were defined as p-tau > 27 pg/ml (T +) and t-tau > 300 pg/ml (N +) (Blennow et al., 2019). In the AD subjects, A/T/N (+/+/+) group accounted for 68.8% (11/16) and A/T/N (+/−/−) group accounted for the rest. Among the MCI subjects, A/T/N (+/+/+) group accounted for 42.3% (11/26), A/T/N (+/−/−) group accounted for 50% (13/26), and A/T/N (+/+/−) group accounted for the rest. In the CU subjects, each A/T/N (+/+/+) and A/T/N (+/−/−) group accounted for 45% of the total, and A/T/N (+/+/−) group accounted for the rest (10%). Therefore, it was confirmed that the AD group showed a relatively large distribution of A/T/N (+/+/+). The average follow-up period was 26 months. In total, 77 subjects (19 AD, 36 MCI, and 22 CU) were available in the longitudinal analysis.

**TABLE 1:** Baseline characteristics of dataset 1 (ADNI) and dataset 2.

**Dataset 1 (ADNI 3)**	**AD (*n* = 25)**	**aMCI (*n* = 42)**	**CU (*n* = 24)**
Demographics			
Age	73.4 (9.0)	73.2 (7.3)	72.3 (5.6)
Sex, F^a^ (%)	9 (36.0)	20 (47.6)	17 (70.8)
Education	15.9 (2.4)	16.1 (2.5)	17.0 (1.9)
ApoE ϵ4 (%)	19 (76.0)	30 (71.4)	14 (58.3)
TIV	1.57 (0.21)	1.54 (0.16)	1.51 (0.12)
Pathologic burden			
Tau deposition (SUVR)^b^	1.30 (0.38)	1.12 (0.20)	1.08 (0.15)
Aß deposition (SUVR)^c^	1.45 (0.20)	1.38 (0.19)	1.21 (0.18)
Cortical thickness (mm)^c^	2.37 (0.11)	2.48 (0.08)	2.51 (0.11)
CSF p-tau (pg/ml)	34.5 (12.7)	36.1 (19.9)	26.9 (12.9)
CSF t-tau (pg/ml)	343.6 (104.3)	341.0 (150.4)	277.7 (97.7)
Cognitive function			
MMSE^c^	22.0 (3.2)	27.3 (2.3)	29.1 (1.4)
Composite (*z*-scores)^c^	−3.67 (3.02)	0.99 (2.25)	2.92 (1.65)
ADNI-MEM (*z*-scores)^c^	−0.90 (0.52)	0.14 (0.55)	1.02 (0.50)
ADNI-EF (*z*-scores)^c^	−1.03 (1.15)	0.34 (0.89)	0.91 (0.73)
ADNI-LAN (*z*-scores)^c^	−0.67 (0.92)	0.40 (0.86)	0.81 (0.62)
ADNI-VS (*z*-scores)^c^	−1.12 (1.12)	0.13 (0.65)	0.19 (0.71)
ADAS-cog 11 (baseline)^c^	21.1 (6.7)	10.2 (4.3)	5.0 (2.6)
ADAS-cog 11 (follow-up)^c^	29.5 (11.3)	13.2 (7.0)	5.5 (2.4)
**Dataset 2**	**AD** **(*n* = 21)**	**aMCI** **(*n* = 32)**	**CU** **(*n* = 34)**
Demographics			
Age^a^	63.2 (11.3)	69.2 (7.2)	68.7 (6.8)
Sex, F (%)	14 (66.7)	23 (71.9)	22 (64.7)
Education	11.6 (4.4)	11.0 (4.3)	10.6 (4.8)
ApoE ϵ4^b^ (%)	9 (42.9)	17 (53.1)	6 (17.6)
TIV	1.35 (0.12)	1.33 (0.13)	1.37 (0.13)
CDR-SB^c^	6.39 (3.98)	1.90 (0.96)	0.31 (0.43)
Pathologic burden			
Tau deposition (SUVR)^c^	1.39 (0.09)	1.32 (0.11)	1.20 (0.10)
Aß deposition (SUVR)^c^	1.44 (0.15)	1.50 (0.14)	1.10 (0.07)
Cortical thickness (mm)^c^	2.29 (0.13)	2.39 (0.11)	2.45 (0.12)
Cognitive function			
MMSE^c^	19.4 (5.0)	24.3 (3.6)	28.6 (1.2)
Composite^c^	31.5 (11.1)	42.8 (9.5)	61.0 (7.1)
Memory^c^	46.1 (13.3)	61.6 (14.6)	107.9 (15.2)
Executive^c^	56.0 (29.8)	75.5 (24.9)	102.3 (17.5)
Language^c^	34.1 (13.8)	37.6 (13.4)	50.8 (5.4)
Attention^b^	8.6 (2.3)	9.3 (2.6)	11.1 (2.8)
Visuospatial^c^	12.8 (11.9)	30.2 (6.2)	33.0 (4.5)
Clinical progression			
Follow-up (month)	25.8 (12.3)	29.1 (9.0)	23.3 (9.1)
Conversion to AD, n (%)	–	17 (53.1)	1 (2.9)
Conversion to aMCI, n (%)	–	–	1 (2.9)

*Values are mean (standard deviation) or number (%). ^*a*^*p* < 0.05; ^*b*^*p* < 0.01; ^*c*^*p* < 0.001, significant between groups. AD, Alzheimer’s disease; aMCI, amnestic mild cognitive impairment; CU, cognitively unimpaired; ApoE ϵ4, apolipoprotein ϵ4 allele; TIV, total intracranial volume; SUVR, standardized uptake value ratio; p-tau, phosphorylated tau; t-tau, total tau; MMSE, Mini-Mental State Examination; ADAS-cog 11, Alzheimer’s Disease Assessment Scale-Cognitive Subscale 11; Composite score, the average score of five domains; CDR-SB, Clinical Dementia Rating Scale-Sum of Boxes.*

In dataset 2, the mean age was lower in the AD group than that in the prodromal AD and CU groups, attributing to the inclusion of 15 early-onset AD. The portion of ApoE ε4 carriers was higher in the AD spectrum than that in the CU. We could not investigate the A/T/N classification of the respective group because the dataset did not contain CSF information. The average follow-up period was 27 months. A total of 61 participants (18 AD, 28 prodromal AD, and 15 CU) had longitudinal MMSE, and 43 subjects (11 AD, 20 prodromal AD, and 12 CU) had follow-up composite score, memory score, and CDR-SB. The total incidence of diagnostic conversion was 28.8%. The conversion rate of prodromal to AD was 53.1%, and the conversion rate of CU to prodromal or CU to AD was 2.9%, respectively.

### Model Construction

In dataset 1, we confirmed the relationship between cognitive performance and each predictor *via* beta values. The linear regression model revealed that composite score was negatively associated with global tau (β_*tau*_ = −6.35) and Aβ deposition (β_Aβ_ = −1.80). The composite score was positively associated with the global cortical thickness (β_thickness_ = 12.85). The *R*^2^ of the model was 0.52 (*F*-test, *p* = 1.49^*^e^–10^, adjusted *R*^2^ = 0.48). There was no multicollinearity among variables (maximum VIF < 1.85). As a result, the estimated composite *z*-scores were significantly correlated with the actual composite *z*-scores using Pearson’s correlation (*r* = 0.72, *p* = 6.51^*^e^–16^). Then, we dichotomized the actual *z*-score using median value (median = 0.819) and investigated whether the estimated *z*-scores predicted actual *z*-scores well. In a discrete ROC curve analysis, the estimated composite *z*-score classified high and low actual *z*-scores well (AUC = 0.83, 95% CI = 0.75–0.92, accuracy = 0.77, sensitivity = 0.83, specificity = 0.71). Through this analysis, we can conclude that the estimated cognitive function from the biological markers, demographics, and genetic factors would be a reasonable estimate of the actual cognitive function. Finally, we calculated the CR marker using the differences between the actual and estimated composite scores.

Using dataset 2, we already validated the suitability of the model (Lee et al., 2019). In brief, the constructed model demonstrated that the composite score was significantly associated with each global AD neuropathology (β_*tau*_ = −18.86, β_Aβ_ = −32.05, and β_thickness_ = 34.11). The *R*^2^ of the model was 0.57 (*F*-test, *p* < 0.00001). There was no multicollinearity within variables (maximum VIF < 1.9). Estimated composite scores were significantly correlated with actual composite scores (*r* = 0.76, *p* = 2.21^*^e^–17^). Also, we dichotomized the actual composite score using median value (median = 47.2) and investigated whether the estimated composite scores predicted high and low composite scores well. In a discrete ROC curve analysis, the estimated composite score classified high and low actual composite scores reasonably well (AUC = 0.92, 95% CI = 0.87–0.98, accuracy = 0.82, sensitivity = 0.77, specificity = 0.86). Then, we calculated the CR marker from the residuals between the actual and estimated composite scores.

### Effect of the Cognitive Reserve Marker on the Relationship Between Pathological Burden and Cognition

In dataset 1, the CR marker was significantly correlated with years of education (*r* = 0.29, *p* = 0.005). Subjects with more years of education had greater CR than individuals with fewer years of education. The positive association between the CR marker and years of education remained even after adjusting for age, sex, and TIV (*t* = 2.94, *p* = 0.004).

**FIGURE 1:**
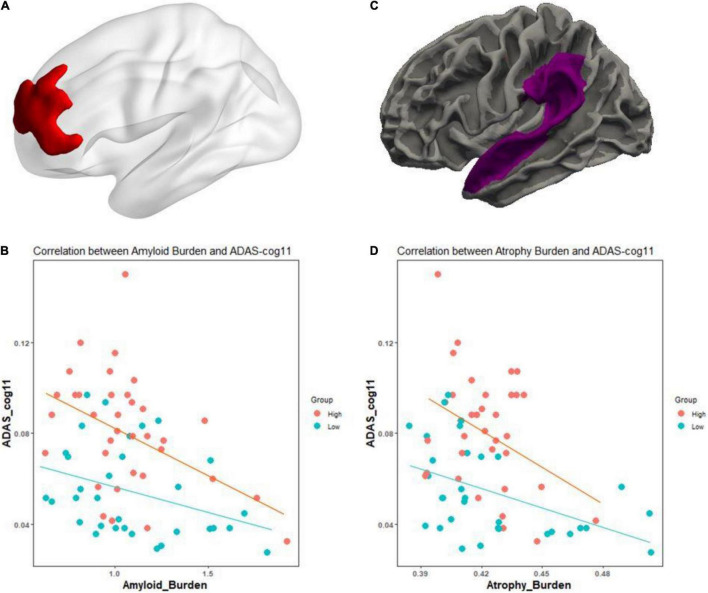
Effect of the CR marker on the relationship between pathological burden and cognitive functions in AD spectrum. **(A)** CR-related regions in amyloid burden using education as a CR proxy (left frontal region). **(B)** Scatterplot for the interaction of the CR marker × left frontal Aβ SUVR on reciprocal of the Alzheimer’s Disease Assessment Scale-Cognitive Subscale 11 (ADAS-cog 11). **(C)** CR-related regions in thickness using education as a CR proxy (left temporoparietal region). **(D)** Scatterplot for the interaction of the CR marker × left temporoparietal atrophy on the reciprocal of the Alzheimer’s Disease Assessment Scale-Cognitive Subscale 11 (ADAS-cog 11). To better represent the effect of CR marker, ADAS-cog 11 score was expressed as reciprocal. For illustration, groups of high and low CR markers (*via* median value) are plotted separately.

Fundamentally, we tested whether the CR marker could modulate the association between AD pathological burden and cognitive function across the AD spectrum. In Figures 1A,B, for Aβ pathology, higher level of education was associated with greater Aβ deposition within the left superior and middle frontal regions, after adjusting for age, sex, Aβ scanner type, and MMSE (*t* = 2.37, *p* < 0.01). We examined whether the CR marker modified the effect of left frontal Aβ burden on cognition in the AD spectrum, controlling for age, sex, and ApoE. There was a significant interaction effect of the CR marker × left frontal Aβ SUVR on ADAS-cog 11 (*t*-stat = 2.17, *p* = 0.034). The interaction effect of CR marker × left frontal Aβ SUVR on MMSE was marginal (*t*-stat = −1.93, *p* = 0.058). As shown in Figures 1C,D, higher education was associated with greater cortical atrophy within the left superior temporal gyrus and supramarginal gyrus after controlling for age, sex, MMSE, and TIV. The interaction effect of the CR marker × left temporoparietal atrophy on ADAS-cog 11 was confirmed to be significant (*t*-stat = 2.29, *p* = 0.026).

### Effect of the Cognitive Reserve Marker on Clinical Progression

Cox regression analysis revealed that in prodromal AD and CU groups, the CR marker was negatively associated with conversion rate, indicating that higher CR was related to lower conversion risk (continuous: HR = 0.57, 95% CI: 0.34 ∼ 0.97, β = −0.56, *p* = 0.037; binary: HR = 0.33, 95% CI: 0.11∼0.97, β = −1.10, *p* = 0.044). The model with the CR marker showed a better model fit than that of the model without the CR marker in a likelihood ratio test (continuous: *p* = 0.031; binary: *p* = 0.037). The model with the CR marker had lower AIC value than that of the model without the CR marker [without CR marker: 115.75, with CR marker: 113.12 (continuous)/113.40 (binary)].

**FIGURE 2:**
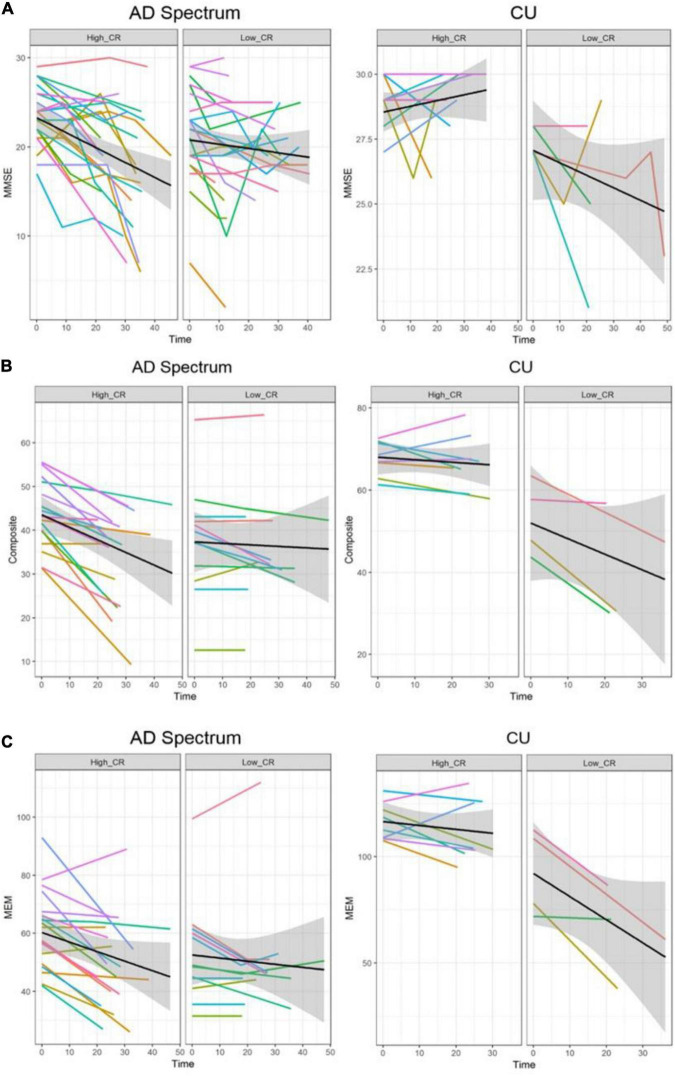
Trajectories of cognitive decline according to the CR marker in AD spectrum and cognitively unimpaired group. Panels **(A–C)** represent the different cognitive scores. In each plot, *X*-axis: Time (month), *Y*-axis: each cognitive score, Left panel: high CR group, Right panel: low CR group. **(A)** Trajectories of MMSE according to the CR marker. **(B)** Trajectories of composite score according to the CR marker. **(C)** Trajectories of memory score according to the CR marker. In the AD spectrum, individuals with high CR showed a steeper decline than the low CR group. In contrast, individuals with high CR showed a attenuated decline than the low CR among the cognitively unimpaired group. Shadows in each plot indicate 95% confidence intervals. CU, cognitively unimpaired group; MMSE, Mini-Mental State Examination; Composite, cognitive composite score; MEM, memory function score.

In the analysis of cognitive decline among AD spectrum, clear cognitive decline was observed across time in all cognitive scores. Particularly, higher CR marker was related to more exacerbated decline in cognitive performance in the AD spectrum (MMSE: *p* = 0.026, composite score: *p* = 0.045, memory score: *p* = 0.036) throughout Table 2 and Figure 2. In CU, cognitive scores decreased over time, with the exception of MMSE. The CR marker also modified the relationship between cognitive function and time. However, the pattern modulated by the CR marker was different from that of the AD spectrum, such that subjects with high CR exhibited attenuated cognitive decline (MMSE: *p* = 0.017, composite score: *p* < 0.001, memory score: *p* = 0.003) throughout Table 2 and Figure 2. The likelihood ratio test revealed that the model with the interaction showed a better model fit than that of the model without interaction in all cognitive scores among CU and AD spectrum. In the aspects of AIC, the model with interaction also had lower AIC values in both AD spectrum and CU as shown in Table 3. AD spectrum participants with high CR exhibited more drastic alterations in disease severity in Table 2. The model with the interaction showed a better model fit than that of the model without interaction among AD spectrum in Table 3.

**TABLE 2:** Effect of the CR marker on cognitive decline and disease severity in AD spectrum and cognitively unimpaired group.

	**AD spectrum**			**CU**		
	**β**	**CI**	***P*-value**	**β**	**CI**	***P*-value**
**MMSE**						
CR marker	1.39	−0.17 ∼ 2.97	0.084	0.89	0.15 ∼ 1.62	0.026
Time	−0.12	−0.16 ∼−0.08	<0.001	−0.02	−0.05 ∼ 0.01	0.26
CR marker × Time	−0.04	−0.08 ∼−0.01	0.026	0.04	0.01 ∼ 0.07	0.017
**Composite score**						
CR marker	5.19	2.20 ∼ 8.21	0.001	5.47	3.25 ∼ 7.65	<0.001
Time	−0.20	−0.28 ∼−0.12	<0.001	−0.18	−0.27 ∼−0.08	0.001
CR marker × Time	−0.08	−0.15 ∼−0.003	0.045	0.20	0.12 ∼ 0.28	<0.001
**Memory score**						
CR marker	7.81	0.35 ∼ 15.32	0.018	8.78	3.91 ∼13.65	0.001
Time	−0.24	−0.37 ∼−0.11	<0.001	−0.47	−0.76 ∼−0.18	0.003
CR marker × Time	−0.14	−0.26 ∼−0.01	0.036	0.39	0.15 ∼ 0.64	0.003
**CDR-SB**						
CR marker	−0.68	−1.58 ∼ 0.22	0.14	−0.10	−0.64 ∼ 0.44	0.72
Time	0.07	0.04 ∼ 0.10	<0.001	0.02	−0.01 ∼ 0.06	0.15
CR marker × Time	0.03	0.01 ∼ 0.06	0.04	−0.02	−0.04 ∼ 0.01	0.23

*AD spectrum, Alzheimer’s disease spectrum; CU, cognitively unimpaired group; MMSE, Mini-Mental State Examination; Composite score, the average score of five domains; CDR-SB, Clinical Dementia Rating Scale-Sum of Boxes; β, Beta coefficient of each variable; CI, 95% confidence interval of the beta coefficient; *P-*value, *p*-value of each variable in the linear mixed model.*

**TABLE 3:** Goodness of fit in models with the interaction between the CR marker and time on cognitive trajectories among AD spectrum and cognitively unimpaired group.

	**AD spectrum**			**CU**		
**Model**	**AIC**	**Log likelihood**	***P*-value**	**AIC**	**Log likelihood**	***P*-value**
**MMSE**						
w/o the interaction	792.07	−385.04	–	147.12	−62.56	–
with the interaction	789.12	−382.56	0.026	143.94	−59.97	0.023
**Composite score**						
w/o the interaction	458.11	−218.05	–	158.54	−70.27	–
with the interaction	456.11	−216.06	0.046	146.99	−63.49	0.0002
**Memory score**						
w/o the interaction	523.29	−250.65	–	198.40	−90.20	–
with the interaction	520.86	−248.43	0.035	191.53	−85.77	0.003
CDR-SB						
w/o the interaction	313.04	−145.52	–	87.57	−34.79	–
with the interaction	310.76	−143.38	0.039	88.09	−34.05	0.22

*AD spectrum, Alzheimer’s disease spectrum; CU, cognitively unimpaired groups; AIC, Akaike Information Criterion; Interaction, interaction of the CR marker with time; w/o, without; MMSE, Mini-Mental State Examination; Composite score, the average score of five domains; CDR-SB, Clinical Dementia Rating Scale-Sum of Boxes.*

More reliable results were obtained from 95% CIs derived from bootstrapping, indicating that the behavioral pattern of CR differed depending on disease stage and was related to accelerated cognitive decline in AD spectrum subjects but with alleviated cognitive decline in CU subjects as shown in Table 4.

**TABLE 4:** Effect of the CR marker on cognitive decline and disease severity (95% confidence intervals from bootstrapping).

	**AD spectrum**	**CU**
	**95% CIs of β values**	**95% CIs of β values**
MMSE		
CR marker	−0.17 ∼ 3.01	0.17 ∼ 1.67
Time	−0.16 ∼−0.08	−0.05 ∼ 0.01
CR marker × Time	−0.08 ∼−0.006	0.01 ∼ 0.07
Composite score		
CR marker	2.00 ∼ 8.50	3.47 ∼ 7.47
Time	−0.28 ∼−0.12	−0.26 ∼−0.09
CR marker × Time	−0.15 ∼−0.007	0.13 ∼ 0.27
Memory score		
CR marker	1.23 ∼ 14.39	4.31 ∼ 13.79
Time	−0.38 ∼−0.11	−0.75 ∼−0.22
CR marker × Time	−0.27 ∼−0.01	0.14 ∼ 0.62
CDR-SB		
CR marker	−1.64 ∼ 0.27	−0.64 ∼ 0.42
Time	0.04 ∼ 0.10	−0.01 ∼ 0.06
CR marker × Time	0.003 ∼ 0.06	−0.05 ∼ 0.01

*AD spectrum, Alzheimer’s disease spectrum; CU, cognitively unimpaired group; MMSE, Mini-Mental State Examination; Composite score, the average score of five domains; CDR-SB, Clinical Dementia Rating Scale-Sum of Boxes; β, beta coefficient of each variable; CI, 95% confidence interval of the beta coefficient.*

In the analysis of ADAS-cog 11 among AD spectrum in dataset 1, the CR marker modified the relationship between cognitive decline and time, such that subjects with higher CR marker exhibited worse cognitive decline (β = 0.07; *p* = 0.026; Supplementary Table 1). Since the higher score in ADAS-cog 11 indicates greater cognitive impairment, AD spectrum subjects with higher CR marker showed more steeper increase in the score. However, in the CU group, the CR marker showed a tendency to modulate the relationship between cognitive function and time, but did not reach a significant level (β = −0.02; *p* = 0.20; Supplementary Table 1). The likelihood ratio test also revealed that the model with the interaction showed a better model fit than that of the model without the interaction in the AD spectrum (Supplementary Table 1).

### Sensitivity Analysis

The constructed model using visual diagnosis of Aβ demonstrated that the composite score was significantly associated with each global AD neuropathology (β_*tau*_ = −18.36, β_Aβ_ = −17.42, β_thickness_ = 22.41). The *R*^2^ of the model was 0.62 (*F*-test, *p* < 0.00001, adjusted *R*^2^ = 0.59). We calculated the CR marker from the residuals between the actual and estimated composite scores. The CR marker correlated well with years of education (*r* = 0.49, *p* < 0.00001). In the analysis of cognitive decline among AD spectrum, the CR marker showed a tendency to modulate the relationship between cognitive function and time, but did not reach a significant level (MMSE: *p* = 0.08, composite score: *p* = 0.13, memory score: *p* = 0.26, Supplementary Table 2). However, subjects with high CR exhibited more drastic alterations in disease severity (CDR-SB: *p* = 0.046). In the longitudinal analysis of CU subjects, the CR marker modulated the association between cognitive performance and time (MMSE: *p* = 0.001, composite score: *p* < 0.001, memory score: *p* = 0.027, Supplementary Table 2). In case of CDR-SB, CU subjects with high CR showed more delayed alterations in disease severity (CDR-SB: *p* = 0.024). Therefore, it was found that although the CR marker using binarization of amyloid PET tended to modulate the relationship between cognition and time to some extent, the CR marker using continuous SUVR value reflects the properties of CR better.

In case of education, years of education was not associated with accelerated cognitive decline (memory score: *p* = 0.12, MMSE: *p* = 0.19, composite score: *p* = 0.78). In the CU group, education modified the relationship between cognitive function and time, such that subjects with higher education exhibited attenuated cognitive decline (memory score: *p* = 0.003, MMSE: *p* = 0.004, composite score: *p* < 0.001, Supplementary Table 3).

We verified whether the CR marker had an independent effect on clinical progression by adjusting global pathological burden as covariates. Even though the values of global pathology were added as covariates, the CR marker still had an effect on clinical progression among AD spectrum in MMSE, memory score, but not composite score (MMSE: *p* = 0.023, memory score: *p* = 0.047, composite score: *p* = 0.06, Supplementary Table 4). The subjects with high CR showed more exacerbated cognitive decline in the AD spectrum. In the analysis of CU subjects, the CR marker also affected the relationship between cognitive function and time (MMSE: *p* = 0.006, memory score: *p* = 0.003, composite score: *p* < 0.001, Supplementary Table 4). In case of CDR-SB, CU subjects with high CR showed more delayed alterations in disease severity (CDR-SB: *p* = 0.047). Through this subsequent analysis, it was determined at least in part that the CR marker could have an additional effect on clinical progression, not simply a derivative of global pathology.

## Discussion

Our major findings were that CR, defined as the difference between actual and estimated cognitive function from overall AD neuropathology, modulated the effect of AD pathological burden on cognition and differentially affected clinical progression depending on the disease status. We demonstrated that the slope of cognitive decline against AD pathological burden was steeper in those with high CR among AD spectrum. We observed that CR affected clinical progression as AD spectrum with high CR exhibited aggravated cognitive decline and disease severity. In contrast, CR was related to mitigated cognitive decline in the CU. These results represent the phenomenon of CR well (Stern, 2009).

Specifically, among CU participants, CR exhibited a protective effect that delayed the onset of cognitive impairment. However, once cognitive decline had commenced, CR was associated with accelerated cognitive deterioration. We can argue this phenomenon as follows (Stern, 2012): Individuals with higher CR may tolerate greater AD neuropathology burden; thus, the point at which cognitive function begins to deteriorate would be delayed relative to those with lower CR. However, there is a certain level (threshold) where the pathological burden is so severe that cognitive function cannot be maintained. Based on this presumption, individuals with higher CR will experience cognitive decline when pathology has progressed to a greater degree and have less time to the end point where pathology defeats cognitive function. This would induce a steeper rate of cognitive decline once it has begun.

Although definitive conclusions on the differential effects of CR depending on disease status have yet to be drawn, multiple studies have reproduced this pattern using various methods to quantify CR. In disease stage showing CR-related accelerated progression, education (Andel et al., 2006; Scarmeas et al., 2006; Hall et al., 2007), occupational complexity (Andel et al., 2006; Boots et al., 2015), IQ (Pavlik et al., 2006; Bracco et al., 2007), and W-score method (van Loenhoud et al., 2019) have been used to identify CR. The literature has reported CR-related attenuated cognitive decline in early stages of AD and CU groups using education (Allegri et al., 2010; Clouston et al., 2019), occupational complexity (Andel et al., 2005; Smart et al., 2014), composite scores (Pettigrew et al., 2017; Soldan et al., 2017), and latent variable method (Reed et al., 2010; Zahodne et al., 2013) to measure CR. However, relatively few studies have been conducted to verify the longitudinal effects of CR on clinical progression across CU and AD spectrum. Most studies have only addressed one specific stage of the disease or unimpaired groups. Moreover, the inclusion of participants was often dependent on clinical entities without AD biomarkers, and results may be misleading. In contrast, our study demonstrated cross-sectional and longitudinal effects of CR on cognitive function in both CU and AD spectrum using an identical method with AD biomarkers.

The underlying mechanism linking CR, AD neuropathology, and cognitive function remains unclear. One tentative theoretical model that integrates CR-related researches has been proposed (Arenaza-Urquijo et al., 2015), whereby neuroprotective and compensatory mechanisms coexist and play differential roles in disease, with neuroprotective mechanisms playing a major role in early stages and compensatory mechanisms coming into play in more advanced disease stages. The concept of neuroprotection in early stages is supported by animal and intervention studies. Animal studies have demonstrated reduced Aβ levels or increased Aβ clearance in mouse with environmental enrichment or voluntary wheel running (Lazarov et al., 2005; Costa et al., 2007). Intervention studies have reported increased perfusion and hippocampal size with exercise and biochemical changes in the hippocampus after cognitive training in the normal elderly people (Valenzuela et al., 2003; Burdette et al., 2010; Erickson et al., 2011). Compensatory mechanisms have been supported by epidemiological, neuroimaging, and autopsy studies (Bennett et al., 2003; Mortimer et al., 2005), showing modulatory effects of CR on the relationship between Aβ pathology and cognition. This differential mechanism of CR is in line with our findings.

Our approach to quantify CR has several strengths over CR proxies. We first attempted to reflect overall AD neuropathology using multimodal neuroimaging, similar to previous residual models, in that CR is defined as a residual. However, our model focused on both structural aspect (neurodegeneration) and proteinopathies (Amyloid and tau). This concept is in line with the latest NIA-AA research framework on the biological definition of AD. Second, our model mirrored the “present” state of CR, unlike one static value of CR proxy for life. As CR is considered an active construct developing from continuous cognitive exposure, our model can be applied dynamically according to the disease stage. Third, we distinguished CR itself from CR proxies, as the latter have inherent limitations such as inter-correlations, static value, and possible mechanisms other than CR, by exhibiting direct impact on the neuropathological process. We believe that the conceptualization of CR without CR proxies can help to avoid these drawbacks.

Understanding the role of CR on the clinical progression in AD has important implications in the clinical and research fields. Despite persistent efforts, disease-modifying treatments have failed for more than three decades. Alternative strategies have been suggested to overcome the failures in disease-modifying therapies for AD. CR may contribute to the development of non-pharmacological approaches for delaying AD onset or promoting AD prevention. Moreover, the concept of CR is closely associated with precision or personalized medicine, an emerging strategy for disease treatment and prevention that takes into account individual variability in genes, environments, and lifestyles. Precision medicine aims to optimize the effectiveness of disease treatment and prevention by considering biological components that may influence disease heterogeneity (Hahn and Lee, 2019). Therefore, considering CR in the clinical environment may provide a basis for accurate prognosis of patients and facilitate an integrated approach. Finally, the genuine effects of an intervention may be identified by categorizing individual patients based on CR levels in clinical trials. Results of the trials may thus be interpreted correctly through the adjustment of baseline differences in CR between groups. Our approach for measuring CR will facilitate understanding of the cognitive trajectory of aging and AD, clarify individuals with susceptibility or resistance to AD pathology, and characterize patients for successful clinical trials.

Our study has some limitations. First, we recognize that our CR model is a relatively simple linear model. We assumed a simple linear relationship between cognitive function and brain pathology and did not consider possible interactions between AD biomarkers or other contributing factors such as white matter hyperintensity or vascular components. In this study, we attempted to extend the validity of our CR model based on primary AD biomarkers (A-T-N) through longitudinal and cross-sectional analysis, rather than aiming to capture the full complexity of CR. Second, the sample size of the longitudinal study was relatively small, and the results may be preliminary. If we applied CSF information rather than PET imaging, it might be possible to obtain a larger size of samples. However, we initially intended to utilize the topological information of amyloid and tau that CSF information cannot provide. Finally, two datasets have heterogeneity in the amyloid positivity of CU individuals. Dataset 2 only contains amyloid-negative subjects due to the scarcity of amyloid positivity in unimpaired states. In Dataset 1 (ADNI), we can utilize amyloid positivity participants in CU individuals. Although the two datasets have heterogeneous characteristics, we considered this to be somewhat meaningful in terms of the opportunity to test the applicability of our methodology.

## Data Availability Statement

The datasets presented in this study can be found in online repositories. The names of the repository/repositories and accession number(s) can be found in the article/Supplementary Material. The ADNI data are accessible from adni.loni.usc.edu/data-samples/access-data.

## Ethics Statement

The studies involving human participants were reviewed and approved by Institutional Review Board of Asan Medical Center and Samsung Medical Center. For ADNI data, ethics approval was obtained by the ADNI investigators. The patients/participants provided their written informed consent to participate in this study. Written informed consent was obtained from the individual(s) for the publication of any potentially identifiable images or data included in this article.

## Author Contributions

DL conceptualized the study, analyzed and interpreted the data, and drafted the manuscript. SS, JR, MO, JO, SO, and JK contributed to the acquisition, processing, and analysis of the data. YJ supervised the study, interpreted the data, and revised the manuscript. All authors contributed to the article and approved the submitted version.

## Conflict of Interest

The authors declare that the research was conducted in the absence of any commercial or financial relationships that could be construed as a potential conflict of interest.

## Publisher’s Note

All claims expressed in this article are solely those of the authors and do not necessarily represent those of their affiliated organizations, or those of the publisher, the editors and the reviewers. Any product that may be evaluated in this article, or claim that may be made by its manufacturer, is not guaranteed or endorsed by the publisher.

## Footnotes

1
https://surfer.nmr.mgh.harvard.edu

2
www.r-project.org
