# Supplementary material for: Effects of Cognitive Reserve in Alzheimer’s Disease and Cognitively Unimpaired Individuals
Source: Front Aging Neurosci. 2022 Feb 7;13:784054. doi: 10.3389/fnagi.2021.784054 (PMC8859488; doi:10.3389/fnagi.2021.784054)
Supplement: Supplementary file 1 [file Data_Sheet_1.docx]

**Supplementary table 1. Effect of the CR marker on cognitive decline and goodness of model fit with the interaction between the CR marker and time according to disease status (ADNI3)**

|  | *AD spectrum* | | | | *CU* | | |
| --- | --- | --- | --- | --- | --- | --- | --- |
|  | *β* | | *CI* | *P value* | *β* | *CI* | *P value* |
| ADAS-cog 11 | | | | | | | |
| CR marker | | -1.33 | -3.84 ~ 1.22 | 0.29 | -0.58 | -1.31 ~ 0.15 | 0.13 |
| Time | | 0.16 | 0.10 ~ 0.21 | <0.001 | 0.01 | -0.02 ~ 0.03 | 0.56 |
| CR marker x Time | | 0.07 | 0.01 ~ 0.13 | 0.026 | -0.02 | -0.04 ~ 0.01 | 0.20 |
|  | |  | *AD spectrum* |  |  | *CU* |  |
| *Model* | | *AIC* | *Log likelihood* | *P value* | *AIC* | *Log likelihood* | *P value* |
| ADAS-cog 11 | | | | | | | |
| w/o the interaction | | 1011.9 | -494.95 | - | 228.18 | -103.09 |  |
| with the interaction | | 1009.0 | -492.47 | 0.026 | 228.59 | -102.29 | 0.20 |

AD spectrum, Alzheimer’s disease spectrum; CU, cognitively unimpaired group; ADAS-cog 11, Alzheimer’s Disease Assessment Scale-cognitive subscale 11; β, Beta coefficient of each variable; CI, 95% confidence interval of the beta coefficient; P value, p-value of each variable in linear mixed model.

**Supplementary table 2. Effect of CR marker using binarization of amyloid value on cognitive decline and disease severity in AD spectrum and cognitively unimpaired group**

|  | *AD spectrum* | | | | *CU* | | |
| --- | --- | --- | --- | --- | --- | --- | --- |
|  | *β* | | *CI* | *P value* | *β* | *CI* | *P value* |
| MMSE | | | | | | | |
| CR marker | | 1.95 | 0.31 ~ 3.63 | 0.02 | 0.75 | 0.16 ~ 1.34 | 0.02 |
| Time | | -0.13 | -0.17 ~ -0.09 | <0.001 | -0.02 | -0.05 ~ 0.006 | 0.13 |
| CR marker x Time | | -0.03 | -0.07 ~ 0.004 | 0.08 | 0.05 | 0.02 ~ 0.08 | 0.001 |
| Composite score | | | | | | | |
| CR marker | | 6.03 | 2.41 ~ 9.81 | 0.002 | 5.34 | 3.42 ~ 7.24 | <0.001 |
| Time | | -0.21 | -0.28 ~ -0.14 | <0.001 | -0.19 | -0.30 ~ -0.08 | 0.002 |
| CR marker x Time | | -0.06 | -0.13 ~ 0.02 | 0.13 | 0.18 | 0.09 ~ 0.27 | <0.001 |
| Memory score | | | | | | | |
| CR marker | | 6.81 | 0.59 ~ 13.01 | 0.03 | 8.07 | 2.83 ~ 13.36 | 0.005 |
| Time | | -0.24 | -0.37 ~ -0.12 | <0.001 | -0.51 | -0.85 ~ -0.17 | 0.008 |
| CR marker x Time | | -0.07 | -0.20 ~ 0.06 | 0.26 | 0.34 | 0.06 ~ 0.62 | 0.027 |
| CDR-SB | | | | | | | |
| CR marker | -0.95 | | -1.85 ~ -0.03 | 0.04 | -0.14 | -0.58 ~ 0.31 | 0.53 |
| Time | | 0.08 | 0.05 ~ 0.10 | <0.001 | 0.03 | 0.002 ~ 0.06 | 0.04 |
| CR marker x Time | | 0.03 | 0.001 ~ 0.06 | 0.046 | -0.03 | -0.05 ~ -0.006 | 0.02 |

AD spectrum, Alzheimer’s disease spectrum; CU, cognitively unimpaired group; MMSE, Mini Mental State Examination; Composite score, average score of five domains; CDR-SB, Clinical Dementia Rating scale sum of boxes; β, Beta coefficient of each variable; CI, 95% confidence interval of the beta coefficient; P value, p-value of each variable in linear mixed model.

**Supplementary Table 3. Effect of years of education on cognitive decline and disease severity in AD spectrum and cognitively unimpaired group**

|  | *AD spectrum* | | | | *CU* | | |
| --- | --- | --- | --- | --- | --- | --- | --- |
|  | *β* | | *CI* | *P value* | *β* | *CI* | *P value* |
| MMSE | | | | | | | |
| Education | | 0.40 | 0.05 ~ 0.74 | 0.02 | 0.18 | -0.02 ~ 0.40 | 0.08 |
| Time | | -0.06 | -0.17 ~ 0.05 | 0.26 | -0.14 | -0.22 ~ -0.06 | 0.002 |
| Education x Time | | -0.006 | -0.02 ~ 0.003 | 0.19 | 0.01 | 0.004 ~ 0.017 | 0.004 |
| Composite score | | | | | | | |
| Education | | 1.06 | 0.31 ~ 1.81 | 0.005 | 1.08 | 0.38 ~ 1.76 | 0.005 |
| Time | | -0.23 | -0.44 ~ -0.02 | 0.03 | -0.72 | -0.92 ~ -0.51 | <0.001 |
| Education x Time | | 0.002 | -0.01 ~ 0.02 | 0.78 | 0.05 | 0.03 ~ 0.06 | <0.001 |
| Memory score | | | | | | | |
| Education | | 0.51 | -0.67 ~ 1.63 | 0.36 | 1.92 | 0.75 ~3.08 | 0.003 |
| Time | | -0.50 | -0.83 ~ -0.17 | 0.004 | -1.56 | -2.24 ~ -0.85 | <0.001 |
| Education x Time | | 0.02 | -0.01 ~ 0.05 | 0.12 | 0.09 | 0.04 ~ 0.15 | 0.003 |
| CDR-SB | | | | | | | |
| Education | -0.19 | | -0.42~ 0.03 | 0.08 | 0.01 | -0.13 ~ 0.15 | 0.90 |
| Time | | 0.06 | -0.02 ~ 0.15 | 0.15 | 0.06 | -0.03 ~ 0.15 | 0.17 |
| Education x Time | | 0.001 | -0.006 ~ 0.008 | 0.73 | -0.003 | -0.01~ 0.003 | 0.31 |

AD spectrum, Alzheimer’s disease spectrum; CU, cognitively unimpaired group; MMSE, Mini Mental State Examination; Composite score, average score of five domains; CDR-SB, Clinical Dementia Rating scale sum of boxes; β, Beta coefficient of each variable; CI, 95% confidence interval of the beta coefficient; P value, p-value of each variable in linear mixed model

**Supplementary Table 4. Effect of CR marker after adjusting global pathology value on cognitive decline and disease severity in AD spectrum and cognitively unimpaired group**

|  | *AD spectrum* | | | | *CU* | | |
| --- | --- | --- | --- | --- | --- | --- | --- |
|  | *β* | | *CI* | *P value* | *β* | *CI* | *P value* |
| MMSE | | | | | | | |
| CR marker | | 2.29 | 1.02 ~ 3.51 | <0.001 | 0.86 | 0.14~ 1.58 | 0.02 |
| Time | | -0.13 | -0.17 ~ -0.09 | <0.001 | -0.03 | -0.06 ~ 0.007 | 0.11 |
| CR marker x Time | | -0.04 | -0.08 ~ -0.006 | 0.02 | 0.04 | 0.01 ~ 0.08 | 0.006 |
| Composite score | | | | | | | |
| CR marker | | 6.32 | 3.15 ~ 9.34 | <0.001 | 5.09 | 3.70 ~ 6.47 | <0.001 |
| Time | | -0.22 | -0.29 ~ -0.14 | <0.001 | -0.19 | -0.26 ~ -0.12 | <0.001 |
| CR marker x Time | | -0.07 | -0.15 ~ 0.003 | 0.06 | 0.21 | 0.15 ~ 0.27 | <0.001 |
| Memory score | | | | | | | |
| CR marker | | 12.38 | 6.72 ~ 18.17 | <0.001 | 7.16 | 1.51 ~ 12.80 | 0.02 |
| Time | | -0.25 | -0.38 ~ -0.13 | <0.001 | -0.45 | -0.74 ~ -0.16 | 0.004 |
| CR marker x Time | | -0.13 | -0.25 ~ -0.001 | 0.047 | 0.40 | 0.16 ~ 0.64 | 0.003 |
| CDR-SB | | | | | | | |
| CR marker | -0.84 | | -1.80 ~ 0.04 | 0.06 | 0.08 | -0.41 ~ 0.58 | 0.73 |
| Time | | 0.07 | 0.04 ~ 0.10 | <0.001 | 0.03 | 0.007 ~ 0.06 | 0.02 |
| CR marker x Time | | 0.03 | -0.001 ~ 0.06 | 0.053 | -0.02 | -0.04 ~ -0.001 | 0.047 |

AD spectrum, Alzheimer’s disease spectrum; CU, cognitively unimpaired group; MMSE, Mini Mental State Examination; Composite score, average score of five domains; CDR-SB, Clinical Dementia Rating scale sum of boxes; β, Beta coefficient of each variable; CI, 95% confidence interval of the beta coefficient; P value, p-value of each variable in linear mixed model.
